# PoCUS identification of distal biceps tendon rupture: a case report

**DOI:** 10.1186/s12245-024-00598-1

**Published:** 2024-03-12

**Authors:** Noman Ali, Alan Tan, Jordan Chenkin

**Affiliations:** Department of Emergency Medicine, Sunnybrook Health Sciences Centre, 2075 Bayview Ave, Toronto, ON M4N 3M5 Canada

**Keywords:** Biceps tendon, Musculoskeletal injuries, Point-of-care ultrasound

## Abstract

**Background:**

In the Emergency Department (ED), patients may present with various injuries that damage muscles, tendons, ligaments, and bony structures. Fractures, joint dislocations, strains, and sprains are prevalent among them. However, distal biceps tendon ruptures are uncommon.

**Case Report:**

Here, we report a case of a young man presented to the ED with a complaint of left arm pain following a martial arts activity. The diagnosis of distal biceps tendon rupture was made using a point-of-care ultrasound (PoCUS), and an early referral to the orthopedic service was provided.

**Conclusion:**

This case highlights the utility of point-of-care ultrasound in assessing musculoskeletal injuries in the ED. Early incorporation of PoCUS into routine clinical practice can potentially improve the overall care of musculoskeletal injuries.

**Supplementary Information:**

The online version contains supplementary material available at 10.1186/s12245-024-00598-1.

## Background

Injuries to soft tissues, muscles, and bones are common in the emergency department (ED). These may include a wide range of injuries, from fractures and dislocations of bones and joints to strains and sprains of tendons and ligaments [1]. ED physicians diagnose and manage these musculoskeletal injuries with a systematic approach that focuses on pain management, immobilization, and diagnostic imaging to identify the extent of damage [2]. In the last decade, point-of-care ultrasound (PoCUS) has evolved as a valuable tool in the assessment of musculoskeletal injuries. Its advantages arise from its availability, non-invasive aspect, cost-effectiveness, time efficiency, and diagnostic reliability [3]. Several recent studies have demonstrated that PoCUS can effectively identify fractures, exclude ligament and tendon injuries, diagnose joint dislocations, and identify joint effusions in the ED [4, 5].

## Case presentation

**Fig. 1:**
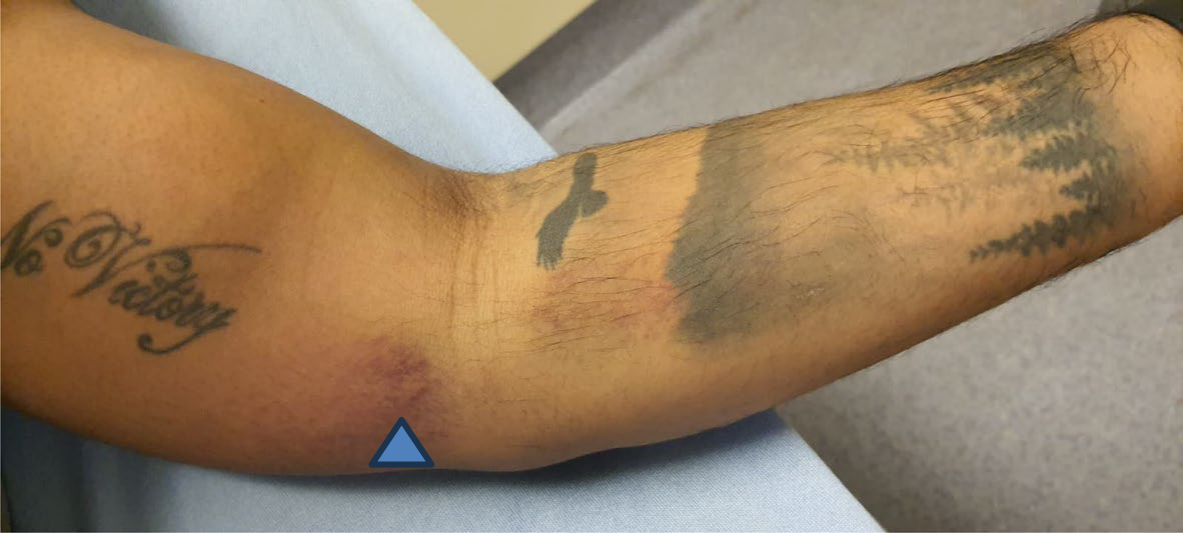
Bruise over the medial aspect of the distal arm and ventral aspect of the proximal forearm (arrowhead)

A 34-year-old man, previously in good health and predominantly right-handed, arrived at the ED with a chief complaint of left arm pain following a martial arts activity. The pain occurred suddenly when his left arm was in a hyper-flexed position at the elbow while being forcefully extended by another individual. Upon examination, vital signs were within normal ranges. The musculoskeletal assessment revealed a semi-flexed position of the left elbow, accompanied by bruising on the medial aspect of the distal left arm and ventral aspect of the proximal forearm (Fig. 1). There was tenderness at the antecubital fossa and the patient was unable to flex his elbow against the resistance. The Hook test was also positive. The distal neurovascular examination was within normal limits.

**Fig. 2:**
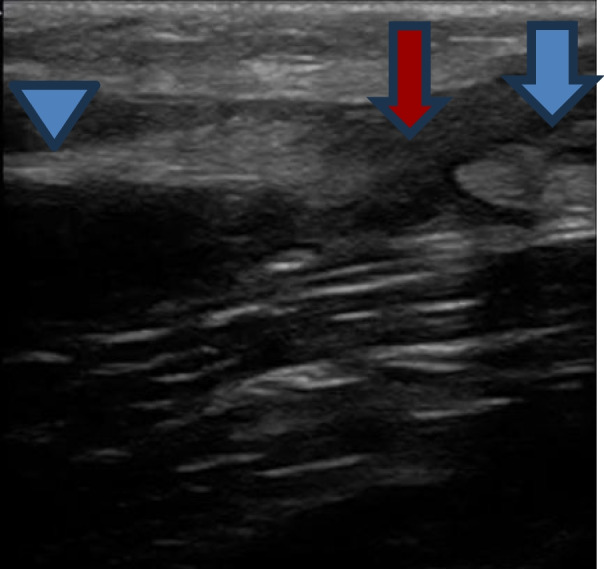
Sagittal sonographic view demonstrating a ruptured distal biceps tendon. Proximal end (blue arrow), distal end (arrowhead), and discontinuous area with surrounding hematoma (red arrow) were visualized

The emergency medicine fellow performed a PoCUS. The patient was positioned supine with his arm semi-flexed. A high-frequency linear array transducer was selected with a musculoskeletal preset. The transducer was placed longitudinally over the ventral surface of the distal left arm to identify the biceps muscle and slid towards the forearm with the marker facing toward the cephalad. The PoCUS showed the disruption of linear tendon fibers with surrounding hypoechogenicity, likely representing blood at the distal musculotendinous junction (Fig. 2, Additional file 1, Video 1). An arm sling was applied, and the patient was discharged on oral analgesics with advice to follow up in the orthopedic clinic. A formal ultrasound appointment was booked for the next day.

A radiology-performed ultrasound confirmed our PoCUS finding. The patient was presented with a choice between conservative (non-surgical) and surgical management based on the confirmed diagnosis. The patient elected to proceed with surgical management. After a week, a surgical procedure was performed to repair the distal biceps tendon rupture.

## Discussion

The distal biceps tendon rupture is infrequent in emergency department presentations, typically resulting from an excessive eccentric force during the extension of the arm from a flexed position [6]. The estimated incidence of distal biceps tendon rupture is 2.55 per 100,000 patient years. The risk of distal biceps tendon rupture is increased by age, smoking, obesity, corticosteroid usage, and overexertion [7].

Diagnosis of distal biceps tendon rupture necessitates a thorough examination, including clinical history, physical examination, and imaging studies. An incorrect diagnosis can result in delayed and ineffective treatment, resulting in ongoing functional impairment, chronic pain, and inadequate recovery [8]. MRI is considered the gold standard for diagnosing distal biceps tendon rupture; however, it is relatively expensive, time-consuming, and not universally available [9].

**Fig. 3:**
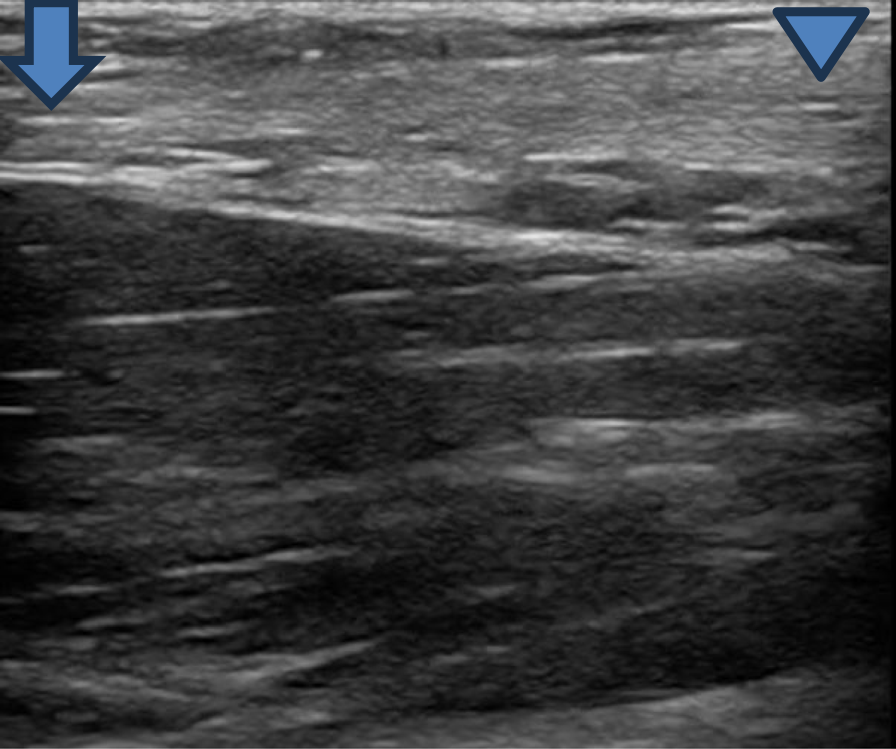
Normal longitudinal view of Sono anatomy of distal bicep tendon. Proximal end (blue arrow) and distal end (arrow head)

PoCUS can be easily performed for the evaluation of distal biceps tendon injuries. The patient should lie sitting or supine with the elbow extended. A high-frequency linear array transducer should be selected with a musculoskeletal preset. The transducer should be placed longitudinally over the distal biceps muscle to identify the musculotendinous junction. Slide the transducer caudally to the insertion at the radial tuberosity. Then the operator should rotate the transducer 90 degrees and evaluate the tendon in the transverse view. The distal biceps tendon should be identified by its well-defined fibrillary appearance (Fig. 3). With a partial tear, the tendon will appear anechoic and the contour will be irregular with peritendinous effusion. In a complete tear, there will be absence of tendon continuity, with retraction of the tendon away from the radial tuberosity accompanied by peritendinous effusion. It is important to include a dynamic assessment by having the patient pronate and supinate as well as flex and extend the elbow [10].

There is growing evidence that PoCUS can be helpful in the diagnosis of biceps tendon injury. A case study by Browning et al. showed the pivotal role of PoCUS in rapidly identifying the rupture of the long head of the biceps tendon [11]. Another study by Javier et al. demonstrated that ultrasound exhibits a statistically significant advantage over MRI in assessing traumatic distal biceps tendon injuries, particularly in detecting complete and high-grade partial tears [10]. While the PoCUS rapidly identified distal biceps tendon rupture in our patient and shortened the length of stay in ED, the patient was booked for a formal ultrasound. In our clinical practice, orthopedic preferences often include obtaining radiology-performed confirmatory imaging.

## Conclusion

In conclusion, this case emphasizes the utility of PoCUS in assessing musculoskeletal injuries in the ED. Its feasibility, cost-effectiveness, and diagnostic accuracy were evident in the early detection of a biceps tendon injury, resulting in rapid diagnosis and appropriate referral for further orthopedic care. PoCUS incorporation into routine clinical practice can potentially improve the overall care of musculoskeletal injuries in the emergency environment.

### Supplementary Information

**Additional file 1. Video1.** Longitudinal view of distal biceps tendon showing disruption of typical linear tendon fibres (sliding probe from medial to lateral).

## Data Availability

No datasets were generated or analysed during the current study.
